# Genetic variants in the NOTCH4 gene influence the clinical features of migraine

**DOI:** 10.1186/1129-2377-14-28

**Published:** 2013-03-26

**Authors:** Elisa Rubino, Pierpaola Fenoglio, Salvatore Gallone, Flora Govone, Alessandro Vacca, Paola De Martino, Maria Laura Giobbe, Silvia Boschi, Lorenzo Pinessi, Salvatore Gentile, Innocenzo Rainero

**Affiliations:** 1 Neurology II, Department of Neuroscience, University of Torino, Via Cherasco 15, Torino, 10126, Italy

**Keywords:** Migraine, Aura, NOTCH4 gene, Polymorphisms, Clinical features

## Abstract

**Background:**

Recent studies suggested an important role for vascular factors in migraine etiopathogenesis. Notch4 belongs to a family of transmembrane receptors that play an important role in vascular development and maintenance. The aim of this study was to test the hypothesis that polymorphisms of the NOTCH4 gene would modify the occurrence and the clinical features of migraine.

**Findings:**

Using a case–control strategy, we genotyped 239 migraine patients and 264 controls for three different non-synonymous polymorphisms (T320A, G835V, R1346P) of the NOTCH4 gene and for the (CTG) n-encoding polyleucine polymorphism in exon 1. Although the analyzed polymorphisms resulted not associated with migraine, the clinical characteristics of our patients were significantly influenced by the different NOTCH4 genotypes. Longer duration of disease and severity of neurovegetative symptoms during headache attacks were associated with the R1346P and G835V polymorphisms, respectively. In female patients, worsening of migraine symptoms at menarche was significantly correlated with T320A polymorphism.

**Conclusions:**

Our study shows that genetic variations within the NOTCH4 gene significantly modify the clinical characteristics of migraine and may have a role in disease pathogenesis.

## Introduction

Migraine is a chronic and disabling neurovascular disorder that affects approximately 15% of the general population in Western Countries [1]. The diagnosis of migraine, as proposed by the International Headache Society (IHS), is mainly based on clinical features such as unilateral throbbing headache, hypersensitivity to sound and light, nausea and/or vomiting and focal neurological symptoms (aura). According to these criteria, migraine is classified into two main subtypes, migraine with aura (MA) and migraine without aura (MO) [2].

Migraine etiology is complex and multifactorial, involving both genetic and environmental factors that interact in a manner yet to be completely understood. Mutations in CACNA1A, ATP1A2 and SCN1A genes have been associated with familial hemiplegic migraine (FHM), a rare monogenic form of migraine [3]. Recently, mutations in the TRESK K2P potassium channel (KCNK18 gene) have been linked to familial migraine with aura [4]. Genetic association studies and genome-wide association studies (GWAS) suggested a role in migraine heritability for a large number of different genes, highlighting the genetic complexity of this disorder [5,6]. However, up to now, no clear genetic association conferring major risk for developing migraine has been found.

Although the pathophysiology of migraine is not fully understood, cortical spreading depression (CSD), a depolarization wave that propagates across the brain cortex and activates the trigeminal nerve in animal models, is speculated to cause the neurological symptoms typical of migraine [7]. It has been suggested that CSD may be initiated by a vascular event, implying that a vascular endothelial dysfunction may increase migraine susceptibility [8]. Genetic studies provided evidence of an increased susceptibility to migraine in patients with polymorphisms in genes associated with endothelial function. The endothelin type A receptor (ETA) gene −231 A/A polymorphism and the Angiotensin I-converting enzyme (ACE) deletion/deletion (DD) polymorphism have been associated with migraine, both with and without aura [9,10]. In addition, genes related to nitric oxide pathway and vascular permeability (like VEGF) resulted significantly associated with both migraine and aura [11].

NOTCH4 gene is located at 6p21.3, in the major histocompatibility complex (MHC), a chromosomal region intensively investigated in migraine [12,13]. The gene encodes for a 2,003 amino acids long protein involved in a cell-cell signalling pathway strongly conserved during evolution. In situ hybridization revealed that NOTCH4 transcripts are primarily restricted to endothelial cells both in embryonic and adult life, suggesting a specific role for Notch4 during vascular development [14]. Mice mutants for the genes of the Notch system show important defect in vascular morphogenesis [15]. In addition, Notch4 plays a crucial role in the differentiation of glial cells, in the organization of neural processes [16] and in the regulation of neural stem cells quiescence, activation, and self-renewal [17].

Notch4 belongs to a family that in humans include four receptors (Notch1-4), binding with different ligands, such as Jagged1, Jagged2, Dll1 and Dll4 [15]. Mutations in the Notch pathway genes, like NOTCH2 and JAG1, are associated with Alagille syndrome, a multisystem disorder that includes cardiac and vascular abnormalities [18]. Mutations in NOTCH3 gene lead to a condition named cerebral autosomal dominant arteriopathy with subcortical infarcts and leukoencephalopathy (CADASIL) that is associated with a higher prevalence of migraine with aura [19].

The aim of the present work was to evaluate the association between migraine and the NOTCH4 gene. We studied several polymorphisms of the gene in a large cohort of Italian migraine patients recruited from a University-based Headache Center and in healthy controls.

## Findings

### Methods

#### Subjects

A group of 239 consecutive unrelated migraine patients (165 females, 74 males; mean age ± SD = 40.6 ± 13.0 yrs), recruited from the Headache Center of the University of Torino (Italy), were selected for the study. The diagnosis of migraine was made, according to the ICHD-II criteria [2], by a neurologist specialized in headaches. One hundred and ninety (79.5%) patients fulfilled the diagnostic criteria for migraine without aura while forty nine patients (20.5%) for migraine with aura. Mean age at onset and duration of the disease were 18.1 ± 9.9 and 22.5 ± 13.5 yrs, respectively. The patients underwent an extensive physical and neurological examination. All the clinical characteristics of the patients were recorded using a standardized questionnaire. Psychological evaluation was performed by a psychologist with the use the Beck Depression Inventory (BDI) and the State and Trait Anxiety Inventory (STAI x-1 and STAI x-2) tests. Table 1 summarizes the demographic and clinical characteristics of migraine patients. A group of 264 age and sex matched healthy subjects (169 females, 95 males, mean age ± SD = 41.6 ± 12.8 yrs) was used as control. The controls were blood donors and were screened to exclude primary headaches. Both patients and controls were of Caucasian origin. Written informed consent was obtained from all participants and the study was approved by the local Ethics Committee. The same population was previously used to perform genetic association studies between DNA polymorphisms and migraine [20,21].

**Table 1:** Demographic and clinical characteristics of migraine patients

	**Migraine patients**		
	**Total**	**Female**	**Male**
Total	239	165	74
Age (mean ± SD)	40.6 ± 13.0	40.7 ± 13.4	40.1 ± 11.9
Age at onset (mean ± SD)	18.1 ± 9.9	18.22 ± 10.1	17.8 ± 9.7
Duration of disease (yrs ± SD)	22.5 ± 13.5	22.6 ± 13.5	22.2 ± 13.6
Attacks/year	56.2 ± 57.9	52.9 ± 53.9	63.6 ± 65.8
Migraine without aura	190	130	60
Migraine with aura	49	30	19
Positive Familial History	184	137	47
Photophobia	192	137	55
Phonophobia	173	122	51
Nausea	184	134	50
Vomiting	121	91	30
Menstrual migraine	116	116	0
STAI-x1	40.4 ± 11.4	41.5 ± 11.6	37.9 ± 10.6
STAI-*x*2	44.2 ± 11.1	44.7 ± 11.2	43.2 ± 10.8
BDI	9.9 ± 7.9	10.4 ± 8.0	8.8 ± 7.6

#### DNA extraction and genotyping

We examined three non synonymous polymorphisms (T320A - rs422951, G835V - rs9267835, and R1346P - rs8192573) and the (CTG) n-encoding polyleucine polymorphism in exon 1 for NOTCH4 gene. All polymorphisms, selected from SNPs database of NCBI (http://www.ncbi.nlm.nih.gov/), are located in regions of putative functional relevance.

Genomic DNA was extracted from 200 μl of peripheral blood using the QIAamp DNA mini Kit (QIAGEN). PCR reactions were performed with 90 ng of genomic DNA, 0.4 unit of Taq Gold DNA polymerase, 250 nM of each primer, 1.5 mM MgCl2 and 50 mM dNTPs. For non synonymous polymorphisms, PCR conditions used were: an initial denaturation at 95°C 10 min followed by 35 cycles 95°C for 1 min, annealing specific temperature for each couple of primers for 1 minute, then at 72°C for 1 min with a final extension at 72°C for 5 min. PCR products were electrophoresed on a 2% agarose TBE 1X gel and stained with ethidium bromide. All non synonymous polymorphisms were analyzed by enzymatic digestion. Genotyping for T320A, G835V, R1346P polymorphisms was performed by digesting PCR products with BsuRI, XmiI, SmaI restriction enzyme, respectively. Conversely, (CTG)n polymorphism was performed on ABI 3700 sequencer (Applied Biosystems, Inc); PCR product was obtained using the same mixture previously described and using the following primers: forward: 5- CCTGCCTGAAGAGGGACAG-3, reverse: 5-CCCCACTGATCATCCTCCTA-3. The forward primer was fluorescently labeled with FAM. Alleles were scored using GeneMarker v1.5 software package (SoftGenetics LLC – http://www.softgenetics.com).

#### Statistics

Fisher’s exact test, *χ*2 test and Monte Carlo Markov chain method (MCMC), were used to compare allelic (AF) and genotypic frequencies (GF) between cases and controls. Patients clinical characteristics were analyzed with ANOVA followed by Bonferroni correction for multiple comparisons. Statistical analysis was performed using Statistical Package for Social Sciences – version 20 (IBM SPSS Statistics 20.0 - August 2011 - SPSS Inc., Chicago Ill). The Hardy-Weinberg equilibrium was verified for observed genotype frequency in all tested populations in order to detect deviation from the expected genotype distribution and to detect genotyping errors. The level of statistical significance was taken at p<0.05.

### Results

Table 2 shows genotypic (GF) and allelic (AF) frequencies of the three non-synonymous coding sequence variants (T320A, G835V, R1346P) of the NOTCH4 gene in migraineurs and controls. GF and AF of the examined polymorphisms were not significantly different between cases and controls. Subsequently, we analyzed the recessive, dominant and additive models for different alleles without reaching any statistical difference.

**Table 2:** **Genotype and Allele Frequencies of the *NOTCH4 *polymorphisms in migraine patients and controls**

	**N**	**GF**			**AF**	
***NOTCH4 *T320A A>G**		**A/A (%)**	**A/G (%)**	**G/G (%)**	**A (%)**	**G (%)**
**- Migraine patients**	239	40 (16.7)	140 (58.6)	59 (24.7)	220 (46.0)	258 (54.0)
**- Controls**	264	67 (25.4)	127 (48.1)	70 (26.5)	260 (49.4)	268 (50.6)
***NOTCH4 *G835V G>T**		**G/G (%)**	**G/T (%)**	**T/T (%)**	**G (%)**	**T (%)**
**- Migraine patients**	239	231 (96.6)	8 (3.4)	0 (0.0)	470 (98.3)	8 (1.7)
**- Controls**	264	256 (97.0)	8 (3.0)	0 (0.0)	520 (98.5)	8 (1.5)
***NOTCH4 *R1346P G>C**		**G/G (%)**	**G/C (%)**	**C/C (%)**	**G (%)**	**C (%)**
**- Migraine patients**	239	223 (93.3)	16 (6.7)	0 (0.0)	462 (96.7)	16 (3.3)
**- Controls**	264	244 (92.4)	19 (7.2)	1 (0.4)	507 (96.0)	20 (4.0)

N= number of subjects, GF = genotypic frequency, AF = allelic frequency.

Table 3 shows allelic frequencies of the (CTG) n-encoding polyleucine polymorphism in cases and controls. No significant difference was found between the two groups.

**Table 3:** **Allelic frequencies of the polymorphic CTG repeat in exon 1 of the *NOTCH4 *gene**

	**Alleles**							
	6R (%)	7R (%)	8R (%)	9R (%)	10R (%)	11R (%)	12R (%)	13R (%)
**Migraine patients**	43 (9.00)	1 (0.21)	3 (0.63)	156 (32.64)	116 (24.27)	112 (23.43)	27 (5.65)	20 (4.18)
**Controls**	53 (10.04)	0 (0.00)	0 (0.00)	167 (31.63)	140 (26.52)	118 (22.35)	40 (7.58)	10 (1.89)

When we divided the migraine patients into different subgroups (MA and MO, episodic and chronic migraine), according to IHCD-II criteria, no significant difference in the distribution of the genetic variants was found.

Finally, we compared the clinical characteristics (age of onset, duration of the disease, frequency of migraine as attacks for year, photophobia, phonophobia, nausea, vomiting, menstrual migraine, and variation at menarche) of our migraine patients according to different NOTCH4 genotypes. Interestingly, we found that the carriage of the allele T of the G835V polymorphism was associated with a higher frequency of vomiting (p=0,034), while carrying the allele C of R1346P polymorphism correlated with a longer duration of disease (p=0,020). When we analyzed our patients according to gender, we found that in females there was a significant association between the allele A of T320A and worsening of symptoms at menarche (p=0,016). When patients were divided into migraine with aura and without aura subgroups, no significant difference was found.

### Discussion

To the best of our knowledge, this is the first study that investigated the association between migraine and the NOTCH4 gene. We found that the analyzed polymorphisms are not significantly associated with the disease. Genotypic and allelic frequencies of the examined polymorphisms were similarly distributed between cases and controls. When patients were divided into migraine with aura and without aura subgroups, no significant difference was found. It is unlikely that genetic variations within the NOTCH4 gene contribute greatly to migraine susceptibility. In addition, we assessed the relation between different NOTCH4 genotypes and the clinical characteristics of migraine. Intriguingly, we found that several characteristics of the disease, like the presence of neurovegetative symptoms, the duration of the disease and the worsening of symptoms at menarche were influenced by NOTCH4 genotypes. Our data support a role for NOTCH4 as a disease-modifier gene in migraine.

There are some limitations in our study that deserve mention and suggest caution in the interpretation of the results. First of all, we performed our study in a sample of patients recruited from an Headache Center that may not be representative of the general migraine population [22]. In addition, the number of migraine with aura patients genotyped in our study may be too low for subgroups analysis. Finally, it is well known that results of genetic association studies must be viewed cautiously until they are confirmed by multiple replications studies [23,24].

Our data are in accordance with several studies suggesting a significant involvement of vascular mechanisms in migraine etiopathogenesis. First of all, migraine symptoms are included in the phenotype of several genetic vasculopathies like cerebral autosomal dominant arteriopathy with subcortical infarcts and leucoencephalopathy (CADASIL), retinal vasculopathy with cerebral leukodystrophy (RVCL) and hereditary infantile hemiparesis, retinal arteriolar tortuosity and leukoencephalopahty (HIHRATL) [25]. It is of interest to note that all the aforementioned disorders are mainly characterized by involvement of small vessels in the brain. Secondly, it is well known that several vascular genetic risk factors, like the methylenetetrahydrofolate reductase (MTHFR) gene and angiotensin I-converting enzyme (ACE) genes, are significantly associated with migraine [26,27]. Finally, epidemiological studies have linked migraine with and without aura to a broader range of ischemic vascular disorders including angina, myocardial infarction, coronary revascularization, claudication and cardiovascular mortality [28].

How genetic and acquired vascular mechanisms might be involved in migraine pathogenesis is still matter of investigation. A dysfunction in endothelial function has been suggested as a key finding in migraine pathogenesis. The endothelium regulates numerous vascular functions. In response to specific stimuli, endothelial cells secrete local vasoactive mediators, including the vasodilator nitric oxide (NO) and vasoconstrictor endothelin-1 (ET-1) and tissue-type plasminogen activator (t-PA). Several observations suggest that endothelial function is abnormal in migraine patients, including an increased prevalence of anti-endothelial cell antibodies that may induce endothelial damage and raised plasma levels of ET-1 and von Willebrand factor [29]. Finally, endothelial progenitor cells were shown to be significantly reduced in migraine patients [30]. An increasing understanding of the role of endothelium dysfunction in migraine pathogenesis may suggest new strategy for disease treatment.

In conclusion, we found no evidence for a strong association between the NOTCH4 gene and migraine and our data does not support the hypothesis that the NOTCH4 gene is a major genetic risk factor for migraine. However, due to the relatively small number of subjects examined, replication studies in larger samples are required in order to confirm our data. Intriguingly, we found that genetic variants within NOTCH4, a gene involved in vascular maintenance and remodeling, significantly influence several clinical features of migraine. Our results further support a role for vascular genetic factor in the disease. Finally, we can hypothesize that the NOTCH4 gene may be particularly involved in some migraine subtypes. Additional studies in families segregating an atypical migraine phenotype are warranted to test this hypothesis.

## Competing interest

The authors declare that they have no conflicting interest.

## Authors’ contributions

ER and IR conceived and supervised the project and drafted the manuscript. PDM, MLG, SG, and SG were responsible for migraineurs patient collection. PF and SB carried out the molecular genetic studies. FG and AV performed the statistical analyses. LP edited the manuscript for intellectual content. All authors read and approved the final manuscript.
